# Characterization of the unique *In Vitro* effects of unsaturated fatty acids on the formation of amyloid β fibrils

**DOI:** 10.1371/journal.pone.0219465

**Published:** 2019-07-10

**Authors:** Miki Eto, Tadafumi Hashimoto, Takao Shimizu, Takeshi Iwatsubo

**Affiliations:** 1 Department of Neuropathology, Graduate School of Medicine, The University of Tokyo, Tokyo, Japan; 2 Department of Lipid Signaling, National Center for Global Health and Medicine, Tokyo, Japan; 3 Department of Innovative Dementia Prevention, Graduate School of Medicine, The University of Tokyo, Tokyo, Japan; H Lee Moffitt Cancer Center and Research Institute, UNITED STATES

## Abstract

Accumulation of amyloid ß (Aß) peptides, the major component of amyloid fibrils in senile plaques, is one of the main causes of Alzheimer’s disease. Docosahexaenoic acid (DHA) is a fatty acid abundant in the brain, and is reported to have protective effects against Alzheimer’s disease, although the mechanistic effects of DHA against Alzheimer’s pathophysiology remain unclear. Because dietary supplementation of DHA in Aß precursor protein transgenic mice ameliorates Aß pathology and behavioral deficits, we hypothesize that DHA may affect the fibrillization and deposition of Aß. Here we studied the effect of different types of fatty acids on Aß fibril formation by *in vitro* Aß fibrillization assay. Formation of amyloid fibrils consists of two steps, i.e., the initial nucleation phase and the following elongation phase. We found that unsaturated fatty acids, especially DHA, accelerated the formation of Aß fibrils with a unique short and curved morphology in its nucleation phase, which did not elongate further into the long and straight, mature Aß fibrils. Addition of DHA afterwards did not modify the morphology of the mature Aß(1–40) fibrils. The short and curved Aß fibrils formed in the presence of DHA did not facilitate the elongation phase of Aß fibril formation, suggesting that DHA promotes the formation of “off-pathway” conformers of Aß. Our study unravels a possible mechanism of how DHA acts protectively against the pathophysiology of Alzheimer’s disease.

## Introduction

Alzheimer’s disease (AD), the major cause of dementia in the elderly, is a progressive neurodegenerative disorder pathologically characterized by the deposition of senile plaques (SPs) and neurofibrillary tangles [1, 2]. Amyloid ß-peptide (Aß), the primary component of amyloid fibrils forming SPs, is proteolytically cleaved as fragments of 38–43 amino acids from Aß precursor protein (APP) by ß- and γ-secretases [1, 2]. The concept that Aß accumulation is the major cause of AD has been supported by several lines of evidence: (i) deposition of Aß42, the most aggregable species of Aß with longer C terminus, is one of the earliest pathological changes observed in the brains of AD patients [3]; (ii) missense mutations of *APP*, as well as those of *PSEN1* and *PSEN2* encoding the catalytic subunit of γ-secretase, altogether cause familial AD [4–7], through a common mechanism of overproduction of Aß(1–42).

*In vitro* studies have shown that the course of Aß fibril formation consists of two steps: a nucleation (lag) phase and the following elongation phase. In the nucleation phase, Aß monomer changes its conformation into ß-sheeted structure and forms an aggregation seed. Once aggregation seeds are formed, soluble Aß monomers associate with the seeds and forms Aß fibrils in the elongation phase [8, 9]. Several factors, including lipids, have been reported to affect Aß aggregation. For example, gangliosides are shown to accelerate Aß aggregation [10, 11], and the effects of lipids to destabilize and resolubilize mature Aß fibers, producing ‘backward’ Aß protofibrils, have also been documented [12].

Several *in vivo* studies in APP transgenic mice have shown that the supplementation of docosahexaenoic acid (DHA, 22:6), an ω-3 fatty acid rich in the brain, ameliorates cognitive dysfunction [13, 14, 15], Aß pathology [13–14, 16–23], or tau pathology [15, 18, 24]. It has also been reported that DHA inhibits *in vitro* Aß aggregation [25, 26], Aß production [16, 22, 27–29], or Aß toxicity in SH-SY5Y cells [26], primary neurons [30] or induced pluripotent stem cells derived from AD patients [31]. These results lead us to speculate that DHA interacts with Aß and affects the process of Aß aggregation, although the mechanisms have remained unclear.

In this study, we have systematically examined the effects of lipids on Aß aggregation, and found that unsaturated free fatty acids, including DHA, promoted the nucleation of Aß aggregation, and that Aß incubated with DHA formed unique short and curved fibrils. We also found that the short and curved Aß fibrils formed with DHA had a lower seeding capacity in Aß fibril formation compared to the authentic mature Aß fibrils. These observations propose a novel mechanism whereby unsaturated fatty acid suppresses Aß aggregation through the formation of “off-pathway” conformers.

## Materials and methods

### Lipids

Dipalmitoyl-phosphatidylcholine (DPPC), palmitoyl-oleoyl-phosphatidylcholine (POPC), palmitoyl-arachidonoyl-phosphatidylcholine (PAPC), pamitoyl-docosahexaenoyl-phosphatidylcholine (PDPC), palmitoyl-oleoyl-phosphatidylethanolamine (POPE), palmitoyl-arachidonoyl-phosphatidylethanolamine (PAPE), palmitoyl-docosahexaenoyl-phosphatidylethanolamine (PDPE), stearoyl-docosahexaenoyl-phosphatidic acid (SDPA), egg phosphatidic acid (PA), and egg phosphatidylglycerol (PG) were obtained from Avanti Polar Lipids (Alabaster, Alabama) (S1 Table).

### In vitro Aß fibril formation

Synthetic Aß(1–40) and Aß(1–42) peptides (PDB 2lfm) were obtained from Peptide Institute, Inc. (Osaka, Japan) or Bachem (Bubendorf, Switzerland). Peptides were solubilized and maintained in 1, 1, 1, 3, 3, 3-hexafluoro-2-propanol (Wako Pure Chemical Industries) at 1 mg/ml, and were passed through a 0.22 μm filter. Before use, peptides were filtered again, dried, and resolubilized in phosphate-buffered saline (PBS) containing 2% (v/v) DMSO at 11 μM concentration for Thioflavin T assays, or 22 μM concentration for the other experiments. Peptides were incubated with lipids or a vehicle control at 37°C at 50 μl aliquots using a PCR thermal cycler.

### Thioflavin T fluorescence assays

Thioflavin T fluorescence assay was performed as previously described [32]. Briefly, the 50 μl aliquots of aggregated Aß(1–40), or Aß(1–42) peptides were mixed with 200 μl of 3 μM Thioflavin T (ThT) in 0.1 M glycine-NaOH (pH 8.5), in a black-bottomed 96-well plate. Fluorescence levels were measured using SpectraMax M2 (λ_ex_ = 443 nm and λ_em_ = 484 nm, Molecular Devices). For each experiment, 3 independent samples were measured.

### Negative stain electron microscopy

Negative stain electron microscopic observation was performed as previously described (32). Briefly, aggregated Aß(1–40) or Aß(1–42) peptides were spread on 400-mesh collodion-coated grids, and were negatively stained with 2% phosphotungstic acid (pH 7.0) (Wako Pure Chemical Industries). Samples were examined with a transmission electron microscope at 80 kV (JEM 1010).

### Cell Culture

Neuro-2a cells, obtained from American type cell culture (CCL-131), were cultured in Dulbecco’s modified Eagle’s medium (Wako Pure Chemical Industries) supplemented with penicillin/streptomycin (Life Technologies) and 10% fetal bovine serum. Cells were grown at 37°C in a humidified atmosphere containing 5% CO_2_.

### Cell toxicity assay

To measure cell viability, 3-(4,5-dimethylthiazol-2-yl)-2,5 diphenyltetrazolium bromide (MTT) assay was utilized. Neuro-2a cells were seeded at a density of 2.5 x 10^4^ cells / 50 μL per well on 96-well plate. After 24 hours, 25 μg/ml of preformed Aß(1–40) fibrils with or without DHA were applied. Cells were cultured for another 24 hrs, 100 μL of 2.5 mg / ml of MTT were applied for 4 hours, and 100 μL of 10% SDS / 0.01M HCl were added to stop the reaction and dissolve the formazan crystals. The intensity of dissolved formazan crystals was measured using SpectraMax M2 at 550 nm.

### Statistical analyses

Results from *in vitro* ThT fluorescence assay were shown as mean ± S.D., and results from MTT assay were shown were shown as mean ± S.E.M.. Quantitative data were analyzed statistically by *t*-test with false discovery rate (FDR) correction, or by one-way ANOVA with post-hoc Tukey’s test using Prism 6 (GraphPad).

## Results

### Unsaturated fatty acids promoted Aß fibril formation *in vitro*

**Fig 1:**
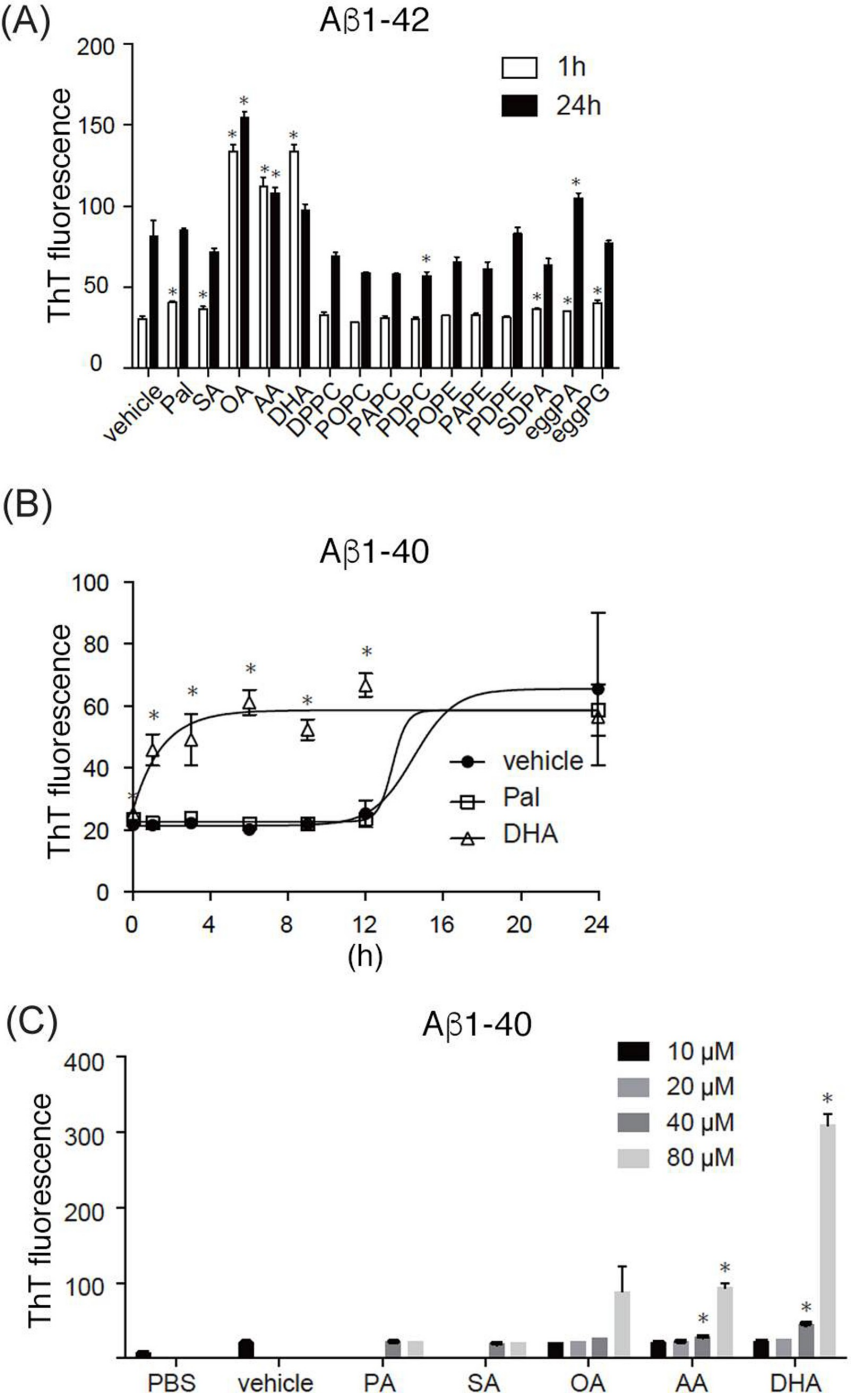
Unsaturated fatty acids promote Aß fibril formation *in vitro*. (A) Fibril formation of Aß(1–42) in the presence of phospholipid liposomes or free fatty acids. 11 μM of Aß(1–42) peptides were incubated at 37°C for 1 or 24 h with 50 μm of different phospholipid liposomes or free fatty acids (PA: palmitic acid, SA: stearic acid, OA: oleic acid, AA: arachidonic acid, DHA: docosahexaenoic acid, DPPC: dipalmitoyl-PC, POPC: palmitoyl-oleoyl-PC, palmitoyl-oleoyl-PC, PAPC: palmitoyl-arachidonoyl-PC, PDPC: palmitoyl-docosahexaenoyl-PC, POPE: palmitoyl-oleoyl-PE, PAPE: palmitoyl-arachidonoyl-PE, PDPE: palmitoyl-docosahexaenoyl-PE, egg PA: egg phosphatidic acid, egg PG: egg phosphatidylglycerol). Representative image out of 4 independent experiments was shown. Error bars show mean ± S.D. Statistics were performed using *t*-test with FDR correction, by comparing with the vehicle-treated samples at the same time period. N = 3, (*) p<0.05, q<0.05. (B) Fibril formation of Aß(1–40) in the presence of free fatty acids. 11 μM of Aß(1–40) was incubated with vehicle, PA or DHA for 0, 1, 3, 6, 9, 12, or 24 h. Error bars show mean ± SD. Statistics were performed using one-way ANOVA with post-hoc Tukey’s test. N = 3. Asterisks show significant differences compared to the vehicle-treated samples. (*) p<0.05. (C) Fibril formation of Aß(1–40) in the presence of different concentrations of free fatty acids. 11 μM of Aß(1–40) was incubated with PA, or SA at 40, 80 μM, and OA, AA, or DHA at 10, 20, 40, or 80 μM. Fibril formation was quantified with ThT. Error bars show mean ± S.D. Statistics were performed using *t*-test with FDR correction by comparing with the vehicle-treated samples. N = 2. (*) p<0.05, q<0.05.

To test whether the unsaturated fatty acids affect the aggregation process of Aß, we first studied the effect of different lipids on Aß fibril formation. Eleven μM of synthetic Aß(1–42) was incubated with 50 μM of different phospholipid liposomes or free fatty acids for 1 h or 24 h at 37°C. As phospholipids, we tested phosphatidylcholine (PC) and phosphatidylethanolamine (PE) with different fatty acid composition (DPPC: 16:0–16:0 PC, POPC: 16:0–18:1 PC, PAPC: 16:0–20:4 PC, PDPC: 16:0–22:6 PC, PAPE: 16:0–20:4 PE, PDPE: 16:0–22:6 PE) (S1 Table). Egg phosphatidic acid (PA), SDPA (18:0–22:6 PA) or egg phosphatidylglycerol (PG) were also used to test the effects of other phospholipids (S1 Table). We also tested the following free fatty acids: palmitic acid (16:0), stearic acid (SA, 18:0), oleic acid (OA, 18:1), arachidonic acid (AA, 20:4) and docosahexaenoic acid (DHA, 22:6) (S1 Table). Aggregation of Aß was quantified with ThT, a fluorescent dye that detects the ß-sheet structures of amyloid fibrils. All phospholipid liposomes we tested showed little or no effect on Aß(1–42) aggregation (Fig 1A). In sharp contrast, we found that unsaturated free fatty acids (OA, AA, and DHA) prominently increased the aggregation of Aß(1–42) at 1 h, which was not further increased until 24 h of incubation (Fig 1A). This suggested that unsaturated fatty acids affected the process of Aß fibril formation. Saturated free fatty acids (palmitic acid and SA) did not show these effects. We further studied the effects of unsaturated free fatty acids on the nucleation as well as the elongation phases of Aß fibril formation using Aß(1–40), which aggregates at a moderate speed. Aß(1–40) was incubated with vehicle, palmitic acid, or DHA for 0, 1, 3, 6, 9, 12, or 24 h to study the time course of aggregation. Aß(1–40) incubated with DHA started to aggregate at very early time points, showing elevated fluorescence at 1 h (Fig 1B). In contrast, Aß(1–40) incubated with vehicle or palmitic acid did not show increased fluorescence until 24 h (Fig 1B). These results suggested that DHA shortened the nucleation phase of Aß(1–40) aggregation. We also studied the concentration dependency of fatty acids on Aß(1–40) aggregation. Aß(1–40) was incubated with palmitic acid, SA, OA, AA, or DHA at 10, 20, 40, or 80 μM. Unsaturated fatty acids showed elevated fluorescence at concentrations higher than 40 μM, and the effect was more striking at 80 μM (Fig 1C). Saturated fatty acids did not show any effects on Aß(1–40) aggregation at all concentrations we used (Fig 1C).

### Unsaturated fatty acids change the morphology of Aß(1–40) fibrils

**Fig 2:**
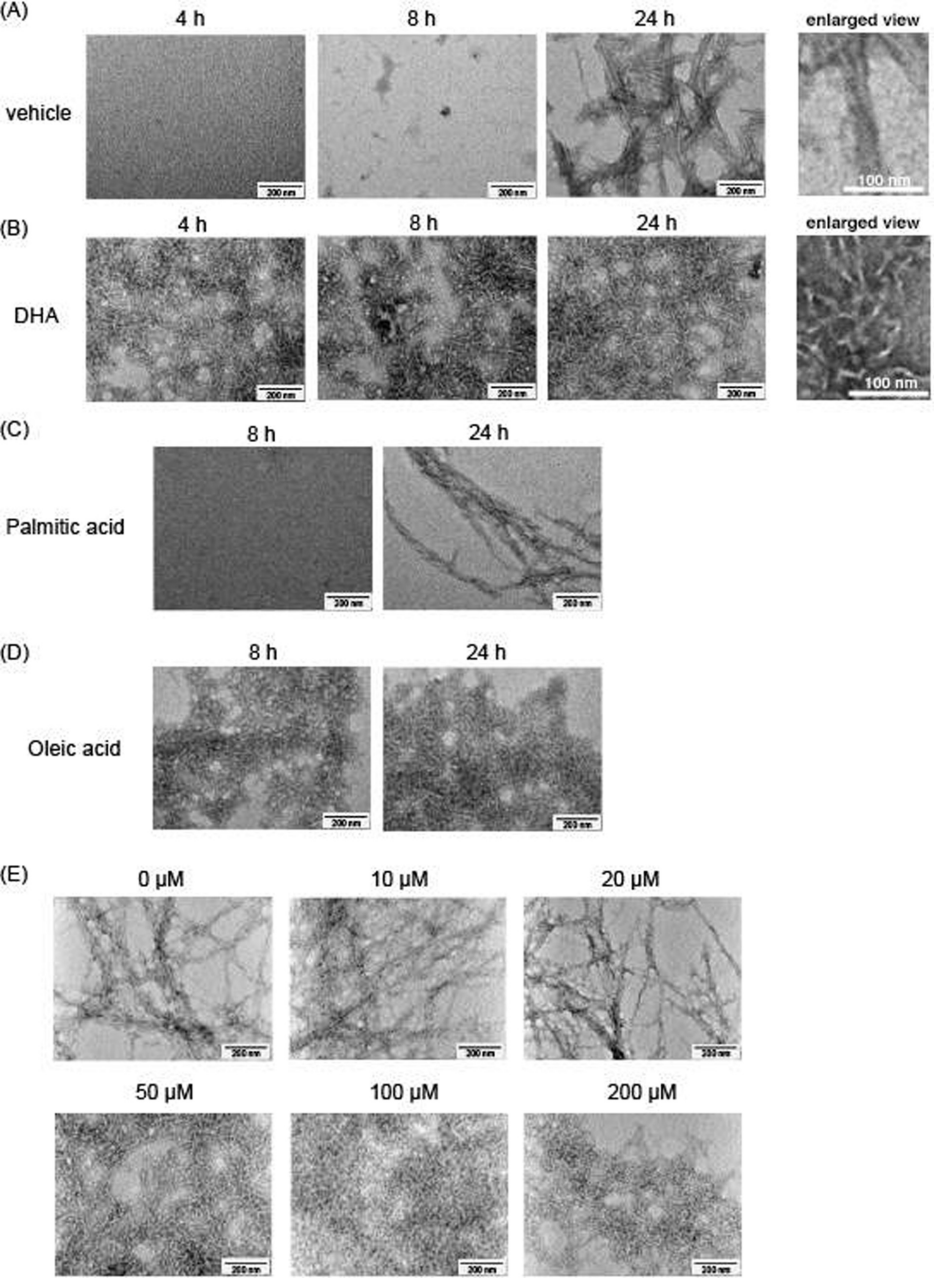
Unsaturated fatty acids change the ultrastructure of Aß(1–40) fibrils. Visualization of the ultrastructure of Aß(1–40) fibrils in the presence of DHA. 22 μM of Aß(1–40) was incubated with vehicle (A) or 50 μM DHA (B) for 4, 8, or 24 h, and negative staining with phosphotanguistic acid was performed to visualize the ultrastructure of fibrils with a transmission electron microscope. 22 μM of Aß(1–40) was incubated with 50 μM palmitic acid (C) or oleic acid (D) for 8 or 24 h. (E) 22 μM of Aß(1–40) was treated with different concentrations of DHA (0, 10, 20, 50, 100, 200 μM) for 24 h.

We next studied whether unsaturated fatty acids affect the morphology of Aß(1–40) fibrils. We incubated Aß(1–40) with vehicle or 50 μM DHA for 4, 8, or 24 h, and performed negative staining to visualize the ultrastructure of Aß fibrils with a transmission electron microscope. Vehicle treated Aß(1–40) did not show any aggregates at 4 and 8 h; however, long, straight fibrils (> 200 nm in length) were observed at 24 h (Fig 2A). DHA treated Aß(1–40) showed fibrils at 4 h, which exhibited short and curved morphology (< 200 nm in length, Fig 2B) compared to the straight fibrils formed upon vehicle treatment. Fibrils with similar morphology were also observed at 8 and 24 h, and straight fibrils did not appear throughout the experiment (Fig 2B). These results suggest that DHA promotes the fibril formation of Aß(1–40), whereas it changes the structure of the fibrils in a way to preclude further elongation into straight fibrils. We also studied the morphologies of Aß(1–40) incubated with 50 μM of palmitic acid or OA. Aß(1–40) incubated with palmitic acid showed similar straight morphology to vehicle-treated Aß(1–40) (Fig 2C), and OA-treated Aß(1–40) showed short and curved morphology similar to DHA treated Aß(1–40) (Fig 2D). These results suggest that the effect of DHA was common to unsaturated fatty acids, not specific to DHA.

We next studied the morphology of Aß(1–40) incubated with different concentrations of DHA (0, 10, 20, 50, 100, 200 μM). At low concentrations of DHA (≤20 μM), DHA treatment had no effect on the morphology of Aß(1–40) fibrils (Fig 2E). At high concentrations (≥50 μM), short and curved structures were observed (Fig 2E). There seemed to be a threshold concentration for DHA to change the state of Aß(1–40) fibrillization, consistent with the results in Fig 1B.

**Fig 3:**
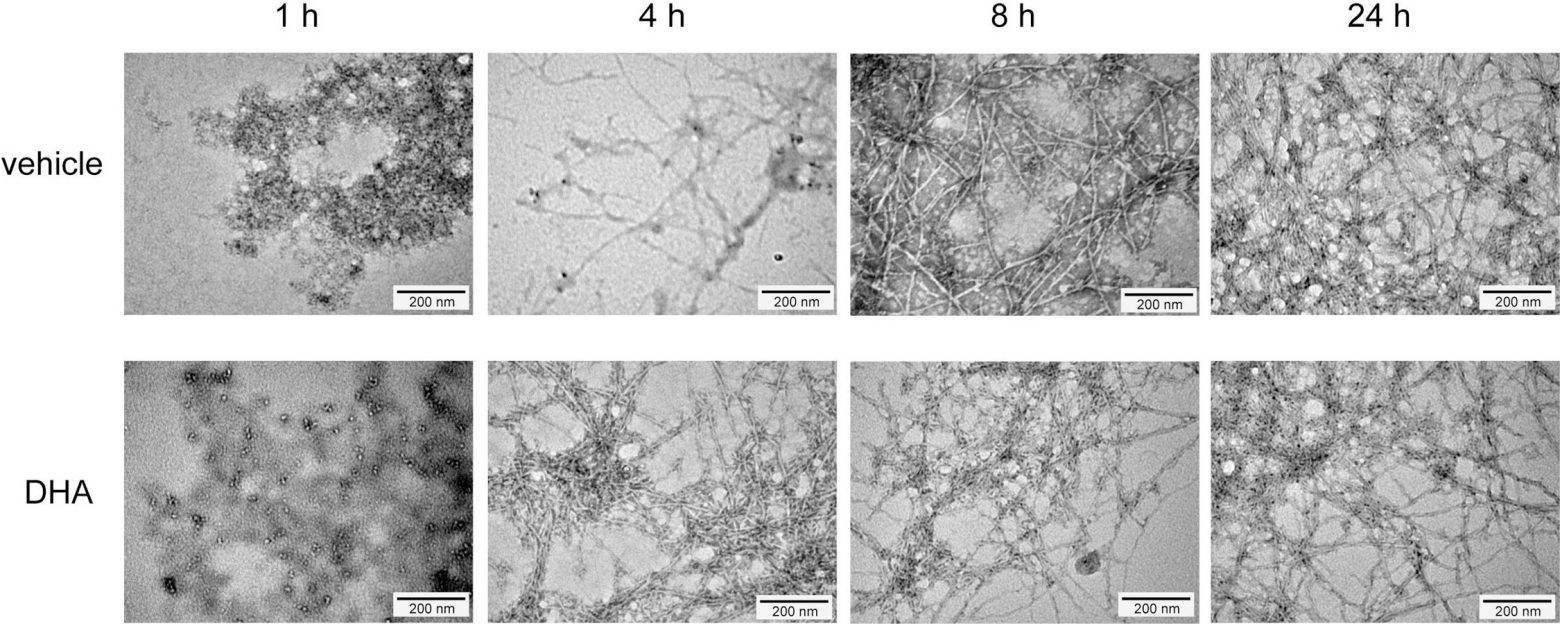
DHA does not change the ultrastructure of Aß(1–42) fibrils. Visualization of the ultrastructure of Aß(1–42) fibrils in the presence of DHA. 22 μM of Aß(1–42) was incubated with vehicle (A) or 50 μM DHA (B) for 4, 8, or 24 h, and negative staining was performed to visualize the ultrastructure of fibrils with a transmission electron microscope.

To see if the change in morphology caused by unsaturated fatty acids is specific to Aß(1–40), we performed similar experiments using Aß(1–42), and have seen that Aß(1–42) incubated with DHA showed straight fibrils similar to vehicle treated Aß(1–42) (Fig 3). Since DHA seemed to accelerate the fibrillization of Aß(1–42) (Fig 1A), too, it was noteworthy that it changed the morphology of only Aß(1–40).

### Oxidation does not alter the effect of DHA against Aß(1–40) fibrillization

**Fig 4:**
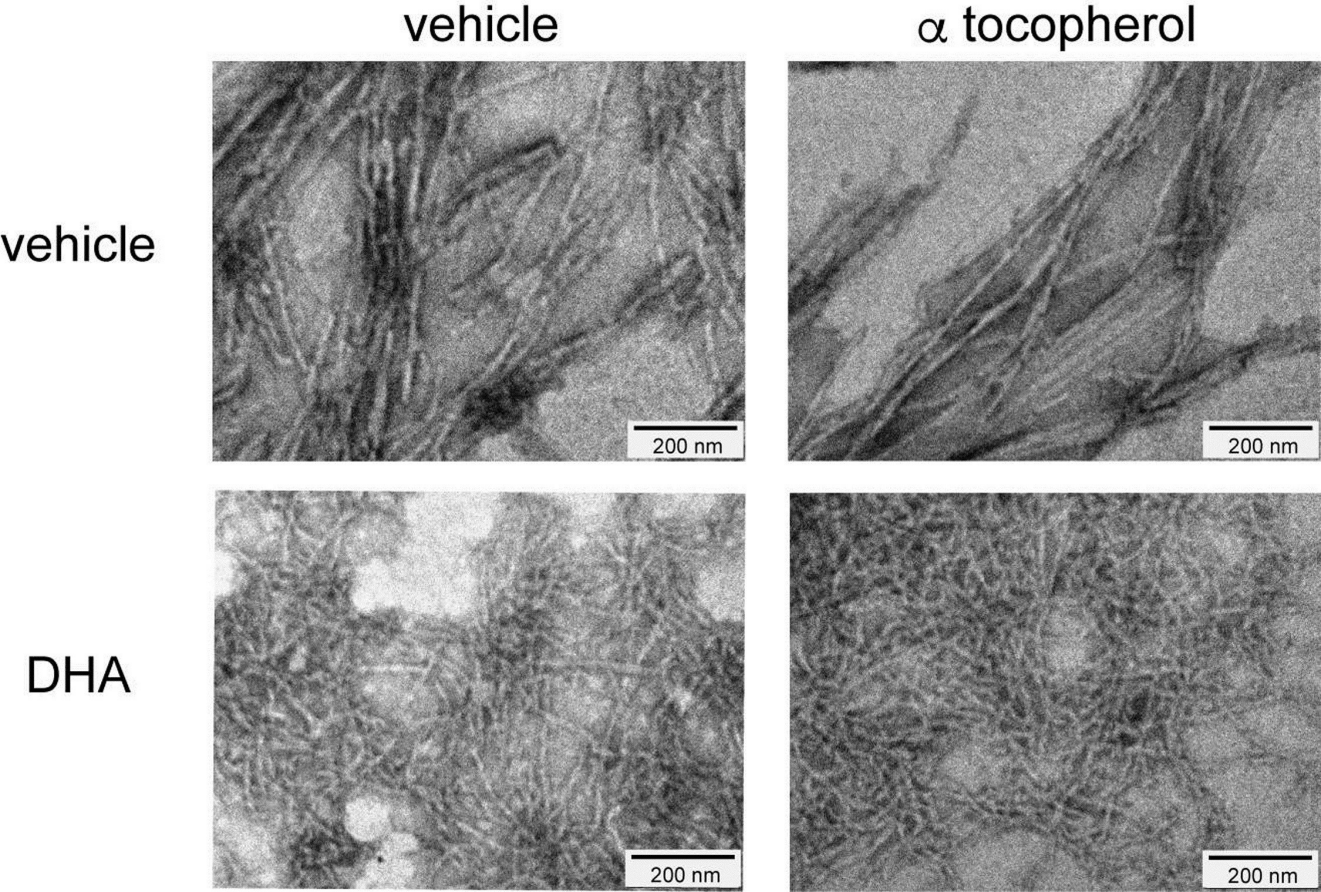
Oxidation does not alter the effect of DHA against Aß(1–40) fibrillization. The ultrastructure of Aß(1–40) fibrils in the presence of DHA with the addition of an antioxidant. 22 μM of Aß(1–40) was incubated with vehicle or 50 μM DHA, either with or without 100 μM α-tocopherol. Negative staining was performed to visualize the ultrastructure of fibrils with a transmission electron microscope.

It is known that unsaturated fatty acids are highly oxidizable. To find whether the effect of DHA on Aß(1–40) fibril formation was caused by oxidation, 100 μM of α-tocopherol, an authentic antioxidant, was added to the mixture of Aß(1–40) and DHA, and incubated at 37°C for 24 h. α-Tocopherol treatment did not alter the effect of DHA against the morphology of Aß fibrils (Fig 4), suggesting that oxidation was not the cause of the effects of DHA on fibril formation.

### Seeding effect of Aß(1–40) fibrils formed in the presence of DHA

**Fig 5:**
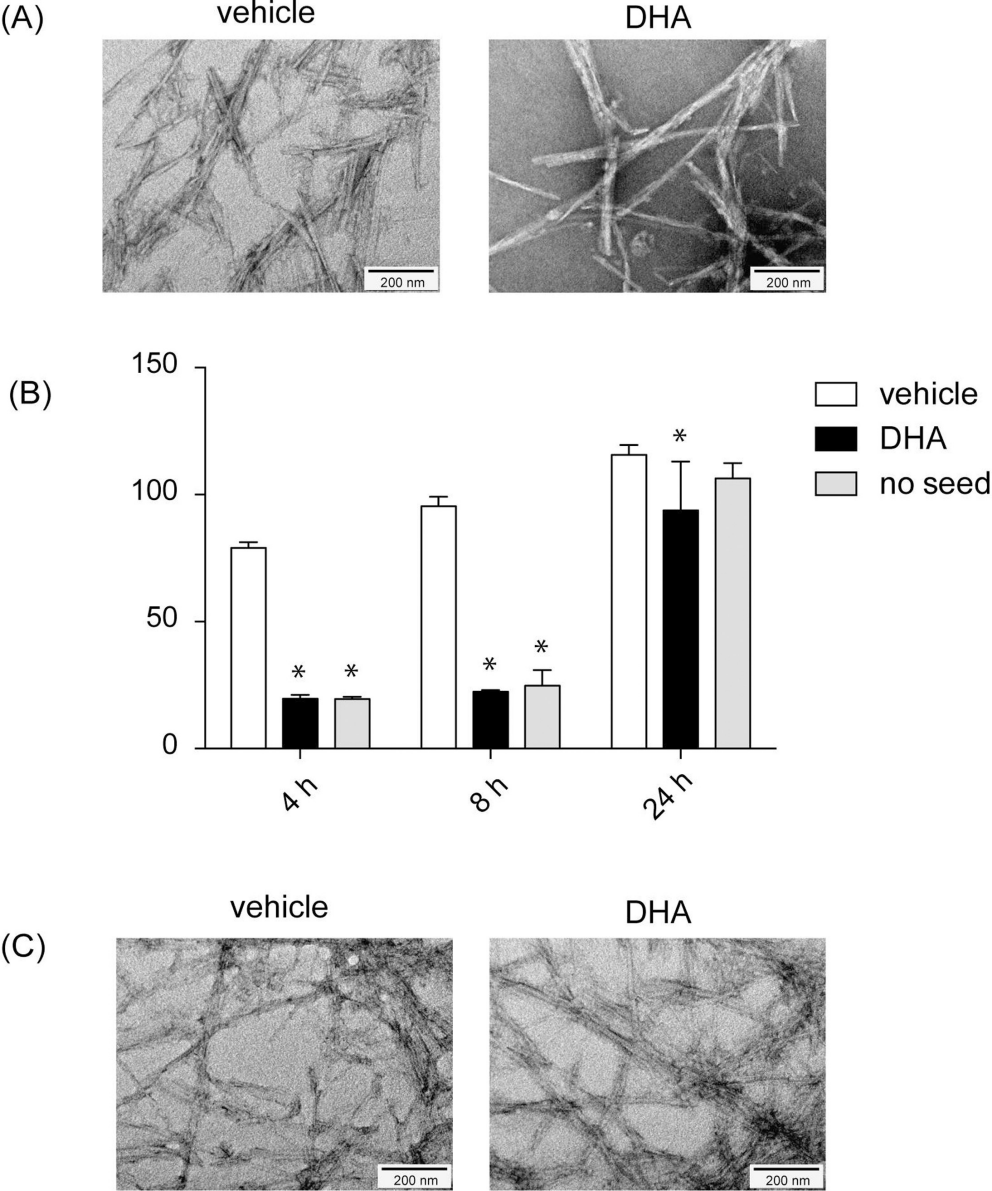
Aß(1–40) fibrils formed in the presence of DHA have lower seeding effect. (A) The ultrastructure of Aß(1–40) incubated with DHA treated Aß(1–40) fibrils as a seed. 22 μM of Aß(1–40) was incubated with vehicle or 50 μM DHA for 24 h, and were added to a new Aß(1–40) mixture at 1/100 dilution as a seed. The new Aß(1–40) mixture was further incubated for another 24 h. Negative staining was performed to visualize the ultrastructure of fibrils with a transmission electron microscope. (B) Fibril formation of Aß(1–40) incubated with DHA treated Aß(1–40) fibrils as a seed. 22 μM of Aß(1–40) was incubated with vehicle or 50 μM DHA for 24 h. Aß(1–40) was added and the mixture was incubated for 4, 8 or 24 h, either with these samples at 1/100 dilution, or without a seed. Aggregation was quantified with ThT. Representative data out of 3 independent experiments was shown. Error bars show mean ± S.D. Statistics were performed using one-way ANOVA with post-hoc Tukey’s test. N = 3, (*) p<0.05. (C) Ultrastructure of Aß(1–40) fibrils with DHA addition after fibril formation. Aß(1–40) was incubated for 24 h, and were further incubated with vehicle or 50 μM DHA for another 24 h.

We found that co-incubation of Aß(1–40) with DHA led to the formation of short and curved fibrils (Fig 2). To see the effect of DHA on pre-formed Aß(1–40) fibrils, we then added DHA to Aß(1–40) fibrils formed by preincubation of Aß(1–40) at 37°C for 24 h, and incubated further for 24 h. Resultant fibrils stayed in the long and straight structure (Fig 5A). The results so far show that DHA treatment does not affect the morphology of the mature fibrils.

Since DHA addition accelerated the nucleation phase of the formation of short and curved fibrils from Aß(1–40), we examined whether the short and curved fibrils made by DHA has a potency to nucleate the fibril formation. To this end, we incubated Aß(1–40) with vehicle or DHA for 24 h to form the straight fibrils or short curved fibrils, respectively, and tested if these preparations serve as aggregation seeds by adding to newly solubilized Aß(1–40) at 1/100 dilution. Contrary to our expectations, however, Aß(1–40) incubated with short curved fibrils further aggregated at a significantly slower rate compared to Aß(1–40) incubated with straight fibrils (Fig 5B). We also observed that the Aß(1–40), incubated with DHA-treated aggregation seeds, formed straight fibrils, but not short curved fibrils (Fig 5C). These findings suggest that the short and curved fibrils have lower seeding capacity than the straight fibrils.

### Toxicity of Aß(1–40) fibrils formed in the presence of DHA

**Fig 6:**
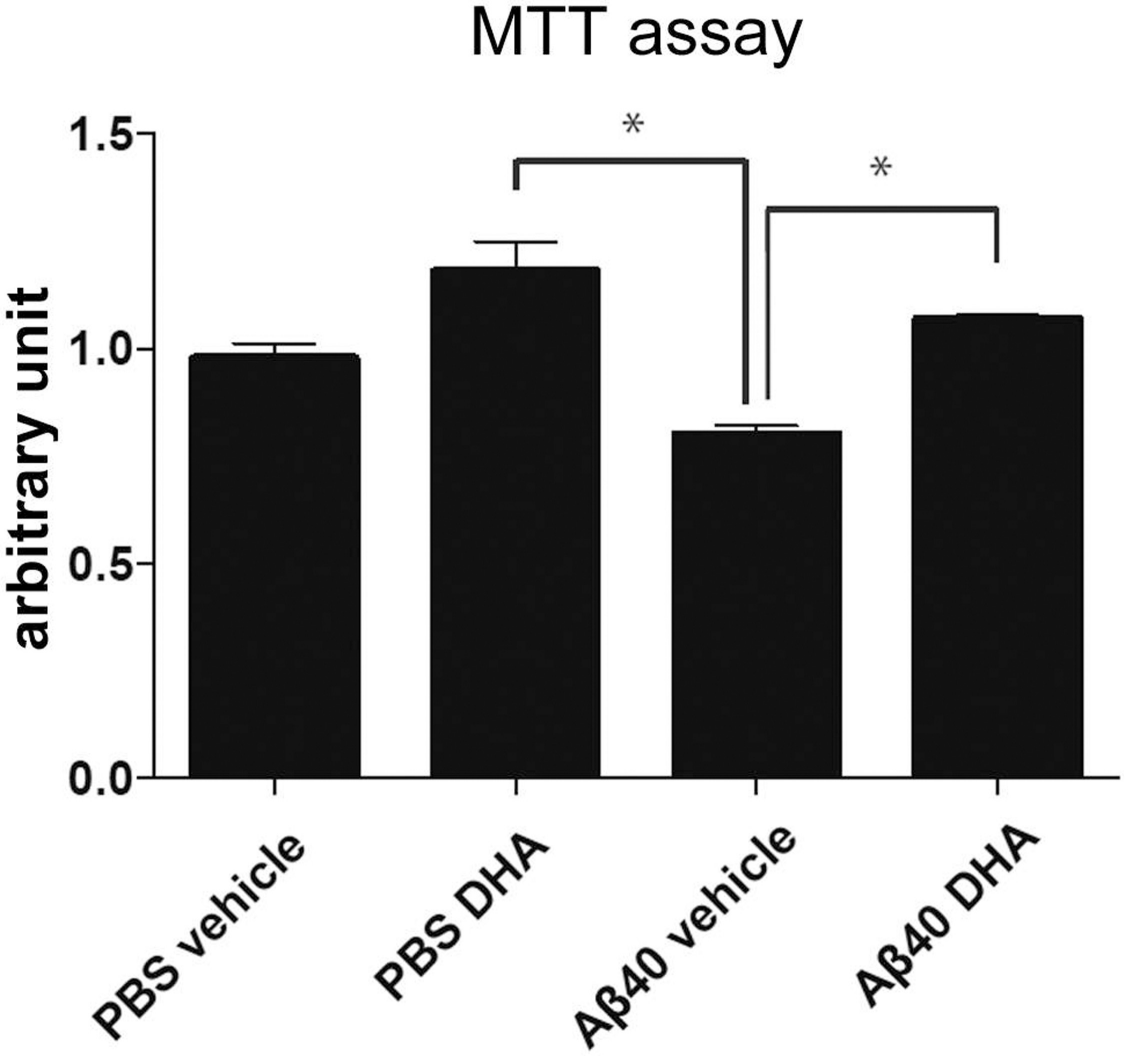
Aß(1–40) fibrils formed in the presence of DHA exhibit lower cytotoxicity. Cytotoxicity of Aß(1–40) fibrils formed in the presence of DHA. 22 μM of Aß(1–40) was incubated with vehicle or 50 μM DHA for 24 h, and these mixtures were added to Neuro-2a cells at 0.025 mg/ml. As a vehicle control for Aß(1–40), PBS or 50 μM DHA was incubated without Aß(1–40) for 24 h and equal volumes of incubates were added to the cells. Cells were cultured for another 24 h, and MTT assays were performed to measure cell toxicity. Graph shows the values of absorbance normalized by the data of the cells treated with PBS control. Error bars show mean ± S.E.M. of three independent experiments. Statistics were performed using one-way ANOVA with post-hoc Tukey’s test. N = 5, (*) p<0.05.

Finally, we examined the toxicity of Aß(1–40) fibrils formed in the presence of DHA. Aß(1–40) incubated with vehicle or DHA for 24 h were added to Neuro-2a cells at 0.0125 mg/ml or 0.025 mg/ml. After 24 h incubation, MTT assays were performed to check the toxicity. Aß(1–40) fibrils formed in the presence of DHA exhibited significantly lower toxicity compared to Aß(1–40) fibrils incubated with the vehicle (Fig 6), suggesting that the short and curved Aß fibrils formed with DHA exerts lower cytotoxicity.

## Discussion

In this study, we found that Aß(1–40) incubated with DHA stayed in a ThT-positive short and curved structure observed at earlier time points (from 4 h), even after incubation for 24 h. The morphology of Aß(1–40) treated with unsaturated fatty acids as short and curved fibrils was similar to that of Aß protofibrils [33, 34]. Aß protofibrils are a metastable intermediate formed in the course of Aß aggregation, which are regarded as “on-pathway” conformers because of their seeding capacity to facilitate formation of Aß fibrils *in vitro* [35] or *in vivo* [36]. Unlike the Aß protofibrils, however, DHA-treated Aß(1–40) was incompetent in elongation of Aß fibrils as aggregation seeds despite their morphological similarity to protofibrils. Thus, we have shown that DHA alters the structure of Aß aggregates in a way to adopt that of an “off-pathway” conformer, which does not further form mature amyloid fibrils, by combining the ultrastructural observation by electron microscopy and quantitative analysis of the dynamics of fibril formation by thioflavin assay.

Several lines of evidence support the existence of off-pathway conformers of Aß: Resveratrol, a red wine polyphenol, remodels Aß oligomers or fibrils into disordered off-pathway conformers [37]. Orcein-related small molecule O4 binds to the hydrophobic region of Aß peptides and converts Aß oligomers into non-toxic, SDS-stable off-pathway aggregates [38]. A cyclic KLVFF-derived peptide, corresponding to the Aß(16–20) region, induces the formation of high-molecular-weight off-pathway Aß oligomers with lower toxicity, which never elongate into fibrils [39]. These results altogether support the notion that a subset of small molecules is capable of remodeling the ß-sheeted structure of Aß oligomers or protofibrils into that of off-pathway conformers.

Our interpretation of the aforementioned results was that DHA induced the formation of off-pathway conformers of Aß, although it is still unclear how DHA alters the structure of Aß aggregates. An *in vitro* study has previously shown that DHA interrupts the microenvironment around the residue Tyr10 of Aß and inhibits the Aß fibril formation [26], suggesting that DHA may change the conformation of Aß through inhibition of the dityrosine cross-link within Aß oligomers or fibrils. The crystal structural analysis or atomistic molecular-dynamics simulations predicted that Aß oligomers interact with lipids and are stabilized in the membrane lipid bilayers [40, 41]. These data suggest that DHA may interact with Aß oligomers and render their tertiary structure into those of Aß aggregates of off-pathway conformers. We found that unsaturated fatty acids including DHA changed the morphology of Aß fibrils into short and curved structure, whereas saturated fatty acids never affected the morphology of Aß fibrils, suggesting that the carbon-carbon double bond in the backbone of fatty acids may be critical in the interaction of unsaturated fatty acids with Aß. Recently, it was reported that APP/PS1 mice fed with unsaturated fatty acid-rich diet deposited less amyloid in brains compared with those fed with saturated fatty acid-rich diet [17]. This suggests that unsaturated fatty acids also affect Aß deposition in brains *in vivo*.

We observed these morphological changes only with Aß(1–40), but not with Aß(1–42), whereas the promoting effect on the nucleation phase was observed both with Aß(1–40) and Aß(1–42). One possible reason for this discrepancy would be that the faster aggregation of Aß(1–42) than Aß(1–40) might have masked the effect of DHA to change the morphologies of Aß(1–42) fibrils. However, we cannot rule out the possibility that Aß(1–42) does not form the morphology of “off-pathway” conformers with short and curved appearance.

We found that phospholipid liposomes, in which unsaturated fatty acid is located at the *sn-2* position, did not affect the speed of Aß aggregation, which prompted us to speculate that unsaturated fatty acids act on the aggregation of Aß in the form of free fatty acids rather than as phospholipid particles. Although the majority of fatty acids in brain tissues are incorporated into membrane phospholipids, it has long been known that free fatty acids are released into the brain parenchyma through hydrolysis of phospholipids by phospholipase A2 [42]. It has been reported that the concentration of free fatty acids in rat brain cortices is ~0.13 μmol/mg lipid phosphorus, and that the percent distribution of the three major free unsaturated fatty acids in the cortices of rat brains are to follow: OA (18:1), AA (20:4), and DHA (22:6) are 15.3%, 13.1%, and 3.9%, respectively [43]; thus, it is possible to speculate that Aß can interact with free unsaturated fatty acids in the brain parenchyma. Previous studies have reported the reduced levels of DHA in the brains of patients with AD patients compared with controls [44–47]. These data suggest that the reduction in the levels of brain DHA may affect the aggregation of Aß, resulting in the acceleration of Aß aggregation. To confirm how DHA acts on Aß *in vivo* in brains, further experiments using animal models would be needed. Because DHA has a long half-life in the brain (~2.5 years [48]), it is difficult to completely eliminate the brain DHA by feeding with DHA-deficient diets. Recently, MFSD2a was identified as a transporter for DHA uptake from blood into the brain, and MFSD2a deficient mice showed decreased DHA levels in the brain [49]; knockout mice of MFSD2a might give us clues to the *in vivo* effects of DHA on the pathophysiology of AD, especially the effect of DHA deficiency on the amyloid pathology.

In sum, we show that unsaturated fatty acid, especially DHA, altered the morphology of Aß(1–40) fibrils into unique short and curved fibrils with reduced potency as aggregation seeds. These findings provide us with clues to mechanisms whereby DHA acts to protect against AD, and might help to develop novel therapeutic strategies against AD.

## Supporting information

S1 File Dataset of the experiments. All relative raw data in this manuscript.
(XLSX)

S1 Table Structures of lipids. Structures of lipids used for the experiments in this study.
(TIF)
