# Supplementary figures and images for: Characterization of the unique In Vitro effects of unsaturated fatty acids on the formation of amyloid β fibrils
Source: PLoS One. 2019 Jul 10;14(7):e0219465. doi: 10.1371/journal.pone.0219465 (PMC6619765; doi:10.1371/journal.pone.0219465)

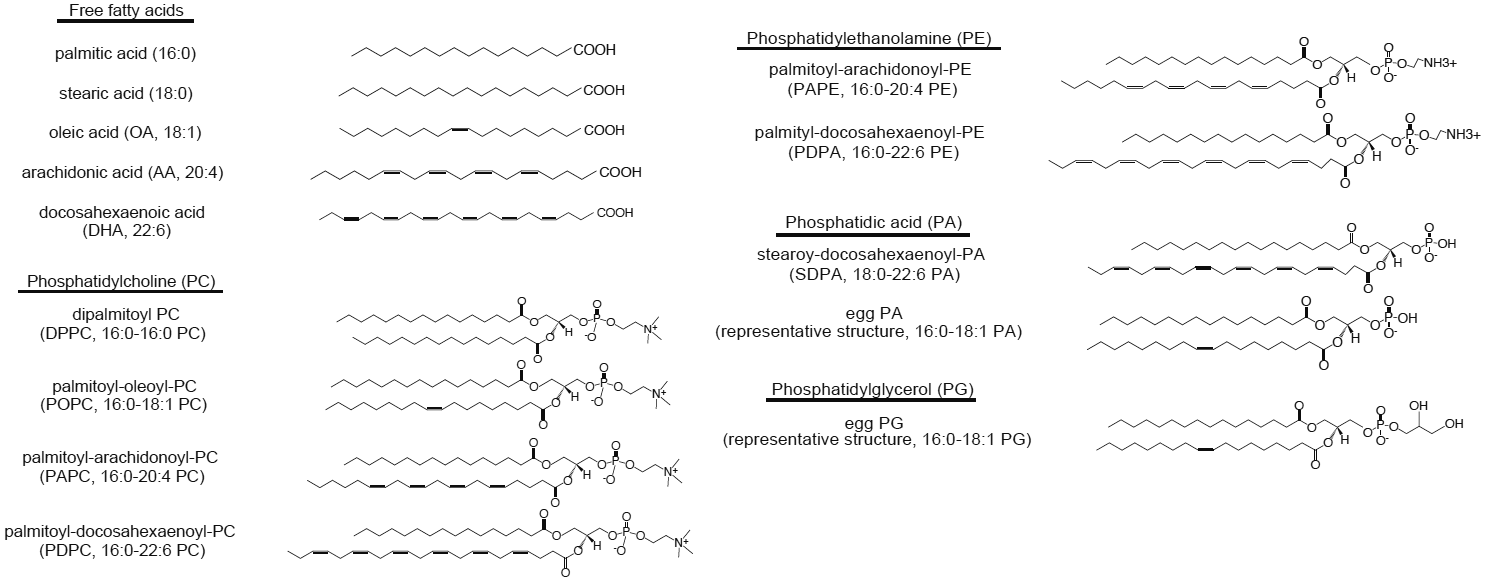

Supplement: S1 Table — Structures of lipids used for the experiments in this study. (TIF) [file pone.0219465.s002.tif]
